# Newborns bilirubin concentration determined by different methods in relation to hematocrit and albumin level

**DOI:** 10.2478/jomb-2019-0030

**Published:** 2020-01-23

**Authors:** Joanna Berska, Jolanta Bugajska, Krystyna Sztefko

**Affiliations:** 1 Jagiellonian University College of Medicine, Clinical Biochemistry Department, Institute of Pediatrics, Krakow, Poland

**Keywords:** albumin, bilirubin, diazo method, hematocrit, newborns, vanadate oxidation method, metoda oksidacija vanadata, novorođenčad, hematokrit, diazo metoda, bilirubin, albumin

## Abstract

**Background:**

Monitoring of bilirubin is essential during early neonatal life. Bilirubin in high concentration is toxic to the brain and might cause irreversible neurological damage. Several different methods for bilirubin determination are available nowadays, but inconsistent results may be obtained. The study aimed to compare dry chemistry methods with vanadate oxidation method for bilirubin determination in relation to hematocrit and albumin level in neonates and infants.

**Methods:**

The study included 98 consecutive serum samples from newborns and infants (47 boys and 51 girls, mean age 19 ± 15 days) treated in the University Children's Hospital in Krakow. Total bilirubin (TBil) and neonatal bilirubin (NBil) concentration were measured by dry chemistry analyser (Vitros 4600, Ortho Clinical Diagnostics Inc.). Total bilirubin (TBil_V_) was also measured using vanadate oxidation method (Cormay, Poland). Albumin concentration and blood morphology have been routinely determined in all children.

**Results:**

No significant differences between the mean value of NBil (69.00 ± 67.76 μmol/L), TBil (81.26 ± 70.13 μmol/L) and TBilV (75.90 ± 60.62 μmol/L) were noticed. High coefficient correlation between NBil and TBil as well as between NBil and TBil_V_ were noticed (Pearson's analysis, r = 0.99, r = 0.97, respectively; p < 0.0001 in both cases). There was a positive correlation between the difference (TBil_V_ - NBil) and hematocrit (p < 0.009, r = 0.2664).

**Conclusions:**

In newborns and infants the same method for bilirubin determination should be used when the concentration of bilirubin is monitored. When using vanadate oxidation method for bilirubin determination, hematocrit value should be taken into account when results are interpreted.

## Abstract (sr)

**Uvod:**

Praćenje bilirubina je neophodno tokom ranog neonatalnog perioda. Bilirubin u visokoj koncentraciji je toksičan za mozak i može izazvati nepovratna neurološka oštećenja. Danas imamo nekoliko metoda za određivanje bilirubina, ali se mogu dobiti nedosledni rezultati. Cilj istraživanja bio je da se uporede suve hemijske metode sa metodom oksidacije vanadata za određivanje bilirubina u odnosu na nivo hematokrita i albumina kod novorođenčadi i odojčadi.

**Metode:**

Studija je obuhvatila 98 uzastopnih uzoraka seruma novorođenčadi i odojčadi (47 dečaka i 51 devojčica, prosečne starosti 19 ± 15 dana), koji su lečena u Univerzitetskoj dečjoj bolnici u Krakovu. Ukupni bilirubin (TBil) i koncentracija neonatalnog bilirubina (NBil) mereni su suvim hemijskim analizatorom (Vitros 4600, Ortho Clinical Diagnostics Inc.). Ukupni bilirubin (TBilV) je takođe meren korišćenjem metode oksidacije vanadata (Cormay, Poljska). Koncentracija albumina i morfologija krvi rutinski su određivani kod sve dece.

**Rezultati:**

Nisu uočene značajne razlike između srednje vrednosti NBila (69,00 ± 67,76 mmol/L), TBil (81,26 ± 70,13 mmol/L) i TBilV (75,90 ± 60,62 mmol/L). Uočena je visoka korelacija između NBil i TBil, kao i između NBil i TBilV (Personova analiza, r = 0,99, r = 0,97, redom; p < 0,0001 u oba slučaja). Postoji pozitivna korelacija između razlike (TBilV - NBil) i hematokrita (p < 0,009, r = 0,2664).

**Zaključak:**

Treba korisititi isti metod praćenja koncentacije bilirubina kod novorođenčadi i odojčadi. Kada se koristi metoda oksidacije vanadata za određivanje bilirubina, treba uzeti u obzir vrednost hematokrita pri interpretiranju rezultata.

## Introduction

Monitoring of serum bilirubin concentration is essential during early neonatal life. Approximately 60% of term babies and 85% of preterm babies develop clinically apparent jaundice [1]. Typically bilirubin concentration increases within 96-120 hours after birth, peaks on day 5 to 7 and then decreases. The physiological jaundice is caused by increased foetal haemoglobin catabolism that accompanies an immature liver as well as intensified enterohepatic circulation [2]. Hyperbilirubinemia is the result of an imbalance between the production and excretion of bilirubin by the liver. Neonatal hyperbilirubinemia can also be caused by various types of disorders both acquired and congenital. Accumulation of bilirubin in the skin and mucous membrane causes yellow discolouration of the skin and sclera of the eye. Bilirubin in high concentration is toxic to the brain because bilirubin can penetrate the blood-brain barrier and might cause irreversible neurological damage [3]. It should be remembered that blood free bilirubin level depends not only on albumin concentration but also on albumin binding capacity. Binding affinity of albumin to bilirubin decreases in sepsis; it is increased with the rise of body temperature and depends on the presence of other ligands which can compete with bilirubin for binding sites on albumin [4].

Accurate measurement of bilirubin in blood or serum is very important for both diagnostic purposes and therapeutic monitoring of small babies with hyperbilirubinemia. Currently, most clinical laboratories rely on automated biochemical analysers for bilirubin measurement which use diazo methods with different modifications, enzymatic methods utilising bilirubin oxidase or vanadate to convert bilirubin to biliverdin. Each of these methods has some limitations, and inconsistent results may be obtained. Ameri et al. [5] obtained comparable results of the total and the direct bilirubin using vanadate oxidase method and diazo method. Whereas, Gu et al. [6] showed that the dry chemistry method and vanadate oxidase method differ significantly compared with the diazo method. Vanadate oxidase direct bilirubin methods offer an advantage over diazo methods in terms of less interference by hemolysis and lipemia, as well as more extensive analytical measurement range, which is particularly evident for newborns and infants [7].

Newborns metabolism is very dynamic and continuous changes in concentration of many analytes in plasma during the first two weeks of life are observed leading to differences in the sample matrix. One of the important plasma components influencing plasma free bilirubin concentration is albumin which is also an acute phase protein and frequently is very low in sick babies. Also, due to a wide range of hematocrit [8] seen in newborns plasma volume differs between samples. Many approaches have been suggested to consider albumin concentration when interpreting bilirubin results; no data are available referring to hematocrit concentration when comparing different methods for bilirubin measurement in newborns and infants. The study aimed to compare dry chemistry methods with vanadate oxidation method for bilirubin determination in relation to hematocrit and albumin level in neonates and infants.

## Materials and Methods

The study included 98 consecutive serum samples from newborns and infants (47 boys and 51 girls, mean age 19 ± 15 days) treated in the University Children's Hospital in Krakow. Total bilirubin (TBil) and neonatal bilirubin (NBil) concentration were measured by a dry chemistry analyser (Vitros 4600, Ortho Clinical Diagnostics Inc., Rochester, NY, USA). TBil was measured by the modified diazo method, where after dissociation from albumin all bilirubins (unconjugated bilirubin, conjugated bilirubin, free bilirubin, delta bilirubin) react with the diazonium salt 4-(N-carboxymethylsulfonyl) benzenediazonium hexafluorophosphate. NBil was determined by using BuBc slides, allow measurement of unconjugated bilirubin (Bu) and mono-and diconjugated bilirubin (Bc). Proteins such as haemoglobin and the albuminbound delta bilirubin as well as lipids and lipochromes are retained in the spreading layer of micro-slide. Potentially interfering compounds are optically blocked in the masking layer preventing them from being measured. The NBil is a sum of Bu and Bc, and it does not include delta bilirubin. Total bilirubin (TBil_V_) was also measured using vanadate oxidation method (Cormay, Poland) by the Cobas Bioanalyzer (Roche Diagnostic Systems, Nutley, NJ, USA). In this method, vanadate is used as an oxidising agent and oxidises bilirubin (yellow colour) to biliverdin (green colour), which is measured spectrophotometrically at 420 nm. Albumin concentration and blood morphology have been routinely determined in all children. The study was approved by the Jagiellonian University Bioethics Committee (Protocol No. 1072.6120.25.2019).

### Statistical analysis

The statistical analysis of data was performed using software package STATISTICA 13 (StatSoft Inc., Krakow, Poland) and Microsoft Office Excel 2010. The Shapiro-Wilk test was used to determine the normality of data distribution. The data was expressed as mean values ± standard deviation (mean ± SD). Comparison between groups was performed by using one-way ANOVA test with Tukey post-hoc test. To determine the relationship between the TBil, NBil and TBil_V_, Pearson’s correlation coefficient was applied. To evaluate the difference between the bilirubin results obtained by different methods, the Bland-Altman method was used, using the average difference ± 2SD as the 95% range of compliance for individual measurements. The level of statistical significance was established as p < 0.05.

## Results

**Figure 1:**
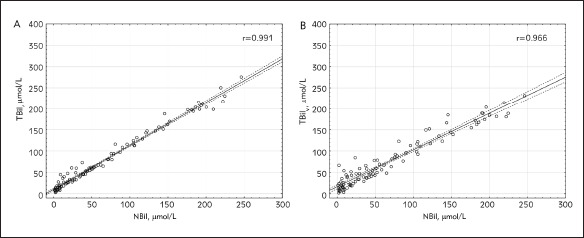
The correlation between NBil and TBil (A) and NBil and TBil_V_ (B). NBil – neonatal bilirubin; TBil – total bilirubin; TBil_V_ – total bilirubin measured by vanadate oxidation method

**Figure 2:**
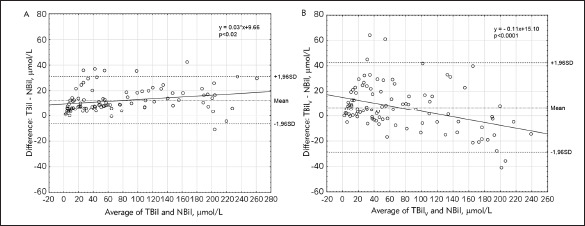
The Bland-Altman plots for the differences between TBil and NBil (A) and TBil_V_ and NBil (B) Solid lines in Figure 2 represent a trend; it is statistically significant (p < 0.0001) only for the difference (TBil_V_ - NBil) and average values between them. (TBil – total bilirubin; NBil – neonatal bilirubin; TBil_V_ – total bilirubin measured by vanadate oxidation method)

**Figure 3:**
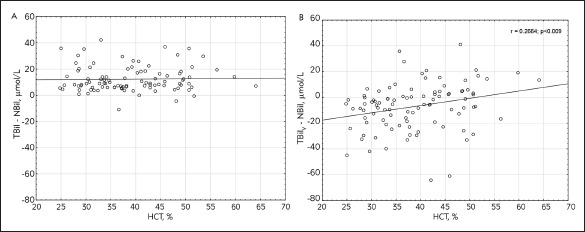
The correlation between the difference (TBil - NBil) and hematocrit (A) and the difference (TBil_V_ – NBil) and hematocrit (B) TBil – total bilirubin; NBil – neonatal bilirubin; TBil_V_ – total bilirubin measured by vanadate oxidation method

**Figure 4:**
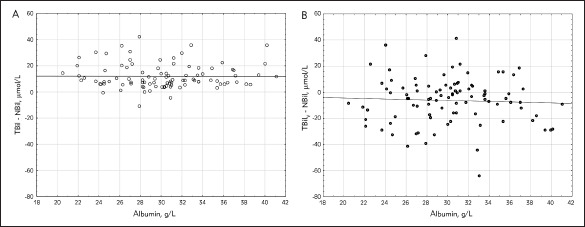
The correlation between the difference (TBil - NBil) and albumin (A) and the difference (TBil_V_ - NBil) and albumin (B) TBil – total bilirubin; NBil – neonatal bilirubin; TBil_V_ – total bilirubin measured by vanadate oxidation method

The characteristic of the study population is reported in Table 1. No significant differences between the mean value of NBil (69.00 ± 67.76 μmol/L), TBil (81.26 ± 70.13 μmol/L) and TBil_V_ (75.90 ± 60.62 μmol/L) were noticed. High coefficient correlation between NBil and TBil as well as between NBil and TBil_V_ were noticed (Pearson's analysis, r = 0.99, r = 0.97, respectively; p < 0.0001 in both cases) Figure 1. As shown in Figure 2, the Bland-Altman plot demonstrated much lower confidence intervals for 95% limits of agreement for the differences between TBil and NBil (-6.57 to 31.07 μmol/L, mean difference 12.25 μmol/L) than the difference between TBil_V_ and NBil (-28.69 to 42.49 μmol/L, mean difference 6.90 μmol/L). The mean hematocrit was 38.8% ± 8.6 %, and the mean albumin concentration was 30.2 g/L ± 4.8 g/L. There was a positive correlation between the difference (TBil V -NBil) and hematocrit (p < 0.009, r = 0.2664) but no correlation between the difference (TBil -NBil) and hematocrit was observed (p = 0.817, r = 0.0238) Figure 3. Also, no correlation between the difference (TBil_V_ -NBil) and albumin level and between the difference (TBil_V _- NBil) and albumin serum level has been observed (Figure 4).

**Table 1:** Characteristics of the study group NBil – neonatal bilirubin; TBil – total bilirubin; TBil_V_ – totalbilirubin measured by vanadate oxidation method

	Mean ± SD
Age of the study group [days]	19 ± 15
NBil, μmol/L	69.00 ± 67.76
TBil, μmol/L	81.26 ± 70.13
TBil_V_, μmol/L	75.90 ± 60.62
Albumin, g/L	30.2 ± 4.8
Hematocrit, %	38.8 ± 8.6
White blood cell count, 10^3^ cells/μL	12.54 ± 5.28
Red blood cell count, 10^6^ cells/μL	3.89 ± 0.72
Haemoglobin, g/L	138.7 ± 47.5
Platelet count, 10^3^ cells/mL	356.95 ± 138.53

## Discussion

According to the Clinical Practice Guideline of American Academy of Pediatrics (AAP), the total serum bilirubin or transcutaneous bilirubin level should be measured in each infant in the first 24 hours of life, to reduce the incidence of severe hyperbilirubinemia [9] However, recent studies have shown that the concentration of serum unconjugated bilirubin is the better indicator of neurotoxicity than the total bilirubin level because only free unconjugated bilirubin can cross the blood-brain barrier and can cause bilirubin encephalopathy [10]. However, no routine method for measurement of the free bilirubin concentration in blood or serum/plasma is available.

Several different methods for bilirubin determination are currently available which do not always yield comparable results and may lead to inconsistent clinical decision. HPLC method is the gold standard for bilirubin measurement but is rarely used due to the high cost of the analysis and expensive equipment. Other methods: diazo methods, enzymatic methods and spectrophotometric methods, are widely used. Analysing the concentration of serum total bilirubin in adults, Apperloo et al. [11] reported a 30% difference between diazo method and spectrophotometric method, but in neonatal specimens, differences were lower 14% to 22%. Total bilirubin can also be measured in whole blood by blood gas analysers or transcutaneously. Borgard et al. [12] compared TBil results obtained using blood gas CO-oximeter module and modified diazo plasma methods in neonatal specimens using different analysers and concluded that methods should be standardised to decrease discrepancy seen between the results. Stephen et al. [13] showed that transcutaneous bilirubinometry overestimates bilirubin level compared to the direct spectrophotometry. Despite this fact, transcutaneous tests are very often the first methods for bilirubin testing, and in case of high bilirubin level, blood is sent to a laboratory for more precise measurements.

In the present study, we compared bilirubin concentrations determined by two different dry chemistry methods and one wet chemistry method. Using dry chemistry technology for neonatal bilirubin measurement, it is possible to precisely and independently measure conjugated and unconjugated bilirubin in serum samples, and the sum of these two forms of bilirubin reflects NBil. Neonatal bilirubin estimated as the sum of Bu and Bc and is equivalent to TBil but only when the delta bilirubin is absent. Technology for measurement of NBil (BuBc slides) was intended for use in neonates [14]. Thus we compared the method for TBil and method for TBil V with the method measuring NBil. In our study, the mean value of neonatal bilirubin (NBil) was lower, but not significantly, compared to TBil and TBil_V_. Similarly, Padmanabhan et al. [15] revealed that in neonatal serum samples, neonatal bilirubin measured using micro slide technology on Vitros 250 was lower than total bilirubin concentration measured using the diazo method by wet technology on Erba-Chem-7. Neonatal serum samples are hemolyzed very often, and haemoglobin interference may be a reason for elevated TBil. Above-cited authors concluded that neonatal bilirubin is a better parameter than TBil in assessing neonatal jaundice in newborns [15]. In another paper, an excellent agreement between neonatal bilirubin (dry chemistry method) and total bilirubin (wet diazo method) [16] has been pointed out. In contrast, Lo et al. [17] showed that neonatal bilirubin values obtained by the Vitros BuBc slides exceeded the values of TBil using the same dry chemistry technology. Furthermore, at low bilirubin concentration, the difference between NBil and TBil was small whereas at a high bilirubin concentration the difference was bigger.

All methods compared in our study were highly correlated. However Bland-Altman plots demonstrated that they are not comparable. In Bland-Altman analysis, the difference between the two methods of measurement is plotted against the average obtained with each of them. The difference between TBil and NBil tends to be higher whereas the difference between TBil_V_ and NBil tends to get smaller as the average increases. Therefore, both methods, TBil and NBil, might be used. TBil method slightly overestimated the results compared to NBil method, but this difference is not clinically relevant. Using the vanadate oxidation method, the higher total bilirubin was, the more significant difference between TBil_V_ and NBil was observed. The level of TBil_V_ was lower than NBil, thus the determination of total bilirubin by vanadate oxidation method may delay the clinical diagnosis and consequently treatment. Likewise, Lano et al. [18] compared methods of bilirubin measurement and revealed the clinically significant variation of the results between methods. They showed that Ortho plasma BuBc results in neonatal specimens were higher than the Roche diazo results and also higher than whole blood CO-oximetry results.

Standard treatment for excessive neonatal hyperbilirubinemia is phototherapy during which bilirubin is converted to more polar photoisomers and can be excreted in bile and urine. It is suggested that hematocrit affects the efficacy of bilirubin photo-alteration because haemoglobin can compete with bilirubin for the light in the spectral range of 410-500 nm. Linfield et al. [19] proved that bilirubin photo alteration is significantly reduced as hematocrit increases. We have investigated whether the level of hematocrit influences the bilirubin determination by wet and dry chemistry methods. The difference between TBil_V_ and NBil increased along with an increase of hematocrit level. The higher hematocrit in the sample means that there is a higher percentage of red blood cells in a given volume of blood but indirectly indicates the higher level of interfering substances, factors that might influence the determination of various analytes. Because each quantitative determination is based on a comparison of the chemical reaction effect in the patient sample and the standard sample, it is important that the matrix of the standard sample is comparable to the patient sample matrix [20]. In the case of bilirubin determination, it is not possible to prepare a standard sample reflecting the heterogeneity and complexity of newborn or infant samples. It can partly explain the discrepancy between the results obtained by different methods.

The low albumin concentration is considered a risk factor for bilirubin neurotoxicity; therefore, measurement of albumin concentration in newborns is recommended by AAP [9]. Albumin in the blood is essential to transport and stabilise bilirubin. If the serum albumin concentration is low, the binding of bilirubin is less efficient, and the risk of kernicterus is increased [21]. The albumin concentration is used to calculate the bilirubin/albumin ratio. Although the bilirubin/albumin ratio is not a better predictor of bilirubin encephalopathy than total serum bilirubin [22], it helps identify infants at risk for neurotoxicity at low total bilirubin due to a low serum albumin concentration [9]. From the analytical point of view, proper management of jaundiced neonates requires improving not only the accuracy of bilirubin measurement but also the accuracy of albumin determination [23]. Imhoff et al. [23] assessed the variability of measurement of bilirubin at various concentrations of albumin in neonates' samples and showed that interlaboratory coefficient of variation of measured bilirubin concentration was strongly dependent on albumin concentration and ranging from 9.2 up to 26%. In the present study, we did not observe the impact of albumin (in concentrations typical for neonates and infants) on bilirubin levels determined by different methods.

However, it is difficult to compare methods for bilirubin determination because different reagents and different calibrators are applied by various manufacturers, methods are prone to interference to various degree, total bilirubin or its fractions can be determined in the whole blood or serum samples and additionally the matrix of the neonates' samples may be very complex. Our results have indicated, that:

- In newborns and infants, the same method for bilirubin determination should be used when the concentration of bilirubin is monitored.

- When using vanadate oxidation method for bilirubin determination, hematocrit value should be taken into account when results are interpreted.

The present study is one of the few studies comparing different methods for bilirubin measurement in newborns and infants. The most commonly used method for bilirubin determination is the diazo method, but due to numerous modifications of this method, sometimes results are not comparable. The message from the present study, particularly important for neonatal care, is: check methods before comparing results of bilirubin and take a closer look at hematocrit level when using vanadate oxidation method for bilirubin determination.

### Conflict of interest statement

The authors stated that they have no conflicts of interest regarding the publication of this article.

## List of abbreviations

NBil, neonatal bilirubin; TBil, total bilirubin; TBil_V_, total bilirubin measured by vanadate oxidation method.
